# Fluoroquinolones for Dermatologists: A Practical Guide to Clinical Use and Risk Management

**DOI:** 10.3390/ph18060800

**Published:** 2025-05-26

**Authors:** Samer Wahood, Omar Alani, Iyla Draw, Lara Shqair, David Wang, Christopher G. Bunick, Giovanni Damiani, Jonathan D. Ho, Sabine Obagi, Hossein Akbarialiabad, Fabrizio Galimberti, Mahmoud Ghannoum, Ayman Grada

**Affiliations:** 1 The Warren Alpert Medical School of Brown University, Providence, RI 02903, USA; samer_wahood@brown.edu; 2 Department of Dermatology, Icahn School of Medicine at Mount Sinai, New York, NY 10029, USA; omar.alani@icahn.mssm.edu (O.A.); lara.shqair@icahn.mssm.edu (L.S.); 3 Department of Dermatology, University of Louisville School of Medicine, Louisville, KY 40202, USA; i0draw01@louisville.edu; 4 Chobanian & Avedisian School of Medicine, Boston University, Boston, MA 02118, USA; dwang2@bu.edu; 5 Department of Dermatology and Program in Translational Biomedicine, Yale School of Medicine, New Haven, CT 06520, USA; christopher.bunick@yale.edu; 6 Department of Dermatology, University of Milan, 20122 Milan, Italy; dr.giovanni.damiani@gmail.com; 7 Departments of Dermatology and Pathology, The University of the West Indies, Mona Campus, Kingston 7, Jamaica; jdho@bu.edu; 8 Department of Dermatology, University of Arizona College of Medicine, Tucson, AZ 85724, USA; sabine@arizona.edu; 9 Department of Dermatology, Spencer Fox Eccles School of Medicine, University of Utah, Salt Lake City, UT 84112, USA; hosseinakbari7575@gmail.com; 10 Department of Dermatology, Conway Medical Center, Conway, SC 29526, USA; fbrzgalimberti@gmail.com; 11 Department of Dermatology, Case Western Reserve University School of Medicine, Cleveland, OH 44106, USA; mag3@case.edu

**Keywords:** fluoroquinolone, skin, dermatology, bacteria, antibiotic stewardship, tendinopathy, safety, skin and soft tissue infections, risk management, toxicity

## Abstract

**Background:** Fluoroquinolones, available in topical and oral formulations, are used to manage bacterial skin and soft tissue infections, including *Pseudomonas aeruginosa*, atypical mycobacteria, and select multidrug-resistant Gram-negative organisms. Their excellent tissue penetration, bactericidal activity, and convenient dosing make them effective for certain skin and soft tissue infections. However, their use is limited by potential safety concerns, including tendinopathy (odds ratio up to 9.1 in corticosteroid users), QT interval prolongation with risk of torsades de pointes, phototoxicity, and rising antimicrobial resistance. **Methods:** A literature search of PubMed, Scopus, and Web of Science was conducted for articles from January 1985 to April 2025 with the search terms (quinolone OR fluoroquinolone) AND (dermatology OR “skin and soft tissue infection” OR “skin structure infection”). Abstracts and presentations were excluded. A Google search used the same terms for articles from government regulatory agencies. **Results:** This review provides practical guidance on the clinical use of topical and oral fluoroquinolones in dermatology. Delafloxacin demonstrated over 90% cure rates in trials for complicated skin infections. However, serious safety concerns remain, including a ninefold increase in tendinopathy risk among older adults on corticosteroids and corrected QT intervals exceeding 500 milliseconds in high-risk patients. Phototoxicity varies, with agents like sparfloxacin linked to heightened ultraviolet sensitivity. Resistance to ciprofloxacin exceeds 20 percent in *Escherichia coli* and *P. aeruginosa* in some populations. Culture-based prescribing, shorter treatment courses, and preference for topical treatments can reduce risk and preserve efficacy. **Conclusions:** Fluoroquinolones remain clinically useful in dermatology when prescribed selectively. Their appropriate use requires careful attention to patient risk factors along with their evolving resistance patterns and ongoing stewardship efforts.

## 1. Introduction

Fluoroquinolones (FQs) are a class of antibiotics that emerged in the 1980s from earlier quinolones, with the addition of a fluorine atom improving their antimicrobial activity, tissue penetration, and dosing efficiency [1,2]. FQs have been instrumental in treating a wide range of infections, including dermatologic conditions such as cellulitis, erysipelas, impetigo, surgical wound infections, and infected diabetic foot ulcers [3]. Their favorable pharmacokinetic properties, including high oral bioavailability and good tissue penetration, have contributed to their popularity in medical therapy.

Despite their widespread clinical use, FQs have been the subject of controversy due to their association with significant adverse effects, including tendinitis and tendon rupture, neuropsychiatric symptoms, and cardiac arrhythmias. As such, careful consideration of their benefits and potential risks is necessary in clinical decision-making [4]. This review is intended to serve as a practical guide for dermatologists by synthesizing current evidence from peer-reviewed studies to clarify the mechanisms of action of FQs, define their clinical indications in dermatologic care, examine associated safety concerns, and present practices for responsible and effective antimicrobial use.

A comprehensive literature search was conducted using PubMed, Scopus, and Web of Science with the search terms (quinolone OR fluoroquinolone) AND (dermatology OR “skin and soft tissue infection” OR “skin structure infection”). Inclusion criteria include peer-reviewed basic science original research articles, clinical guidelines, randomized controlled trials, systematic reviews, review articles, case reports, and case series from January 1985 to April 2025. A Google search used the same search terms with the addition of “site:gov” for articles from United States government regulatory agencies. Abstracts and presentations were excluded. Standardized quality assessments of articles were not performed. No new unpublished data are presented in this review.

## 2. Overview of Fluoroquinolone Antibiotics

FQs have undergone multiple iterations of development, leading to their classification into four generations based on progressive enhancements in pharmacologic properties and the spectrum of activity (Table 1). Nalidixic acid, the prototypical first-generation FQ, demonstrates limited Gram-negative activity and poor tissue penetration, making it unsuitable for most skin infections [5]. Second-generation FQs, including ciprofloxacin and norfloxacin, demonstrate enhanced systemic activity and an expanded spectrum targeting Gram-negative pathogens such as *Escherichia coli*, *Klebsiella pneumoniae*, *Enterobacter cloacae*, *Proteus mirabilis*, *Proteus vulgaris*, *Providencia stuartii*, *Morganella morganii*, *Citrobacter freundii*, and *Pseudomonas aeruginosa*. These agents also exhibit some efficacy against methicillin-susceptible *Staphylococcus aureus* (MSSA) and *Streptococcus pyogenes*. FQs are utilized in the management of both uncomplicated and complicated skin and skin structure infections (SSSIs), including cellulitis and diabetic foot infections [6].

Third-generation FQs, such as levofloxacin and moxifloxacin, exhibit expanded activity against Gram-positive bacteria and atypical pathogens, broadening their efficacy in the treatment of SSSIs, including cellulitis, erysipelas, and surgical wound infections. They demonstrate efficacy against *S. aureus*, including methicillin-susceptible strains, *S. pyogenes*, and *Streptococcus agalactiae*. Additionally, they exhibit activity against Gram-negative pathogens such as *E. coli* and *K. pneumoniae* [3]. Their improved pharmacokinetic profiles facilitate once-daily dosing, enhancing patient adherence to therapy [6,7,8].

Fourth-generation FQs, such as delafloxacin and trovafloxacin, offer enhanced efficacy against resistant Gram-positive pathogens and anaerobic bacteria. Delafloxacin, in particular, has unique changes in chemical structures that differentiate it from other FQs. It has a substituted heteroaromatic ring at the N1 position, which increases its surface area and enhances its antibacterial activity compared to other FQs. It has weak polarity at the C8 position, which is thought to increase effectiveness against quinolone-resistant Gram-positive bacteria. Furthermore, it lacks a basic group at the C7 position, making it weakly acidic and more active in acidic environments like those found in acute bacterial skin and skin structure infections (ABSSSIs) [9]. Fourth-generation FQs are particularly effective against *S. aureus*, including methicillin-resistant *S. aureus* (MRSA), *S. pyogenes*, *S. agalactiae*, *E. coli*, *K. pneumoniae*, and *P. aeruginosa*. Their broad-spectrum activity makes them useful when treating complicated skin and soft tissue infections (SSTIs), especially those involving mixed aerobic and anaerobic pathogens or severe cases such as diabetic foot infections [10,11,12,13].

### 2.1. Mechanism of Action

**Figure 1:**
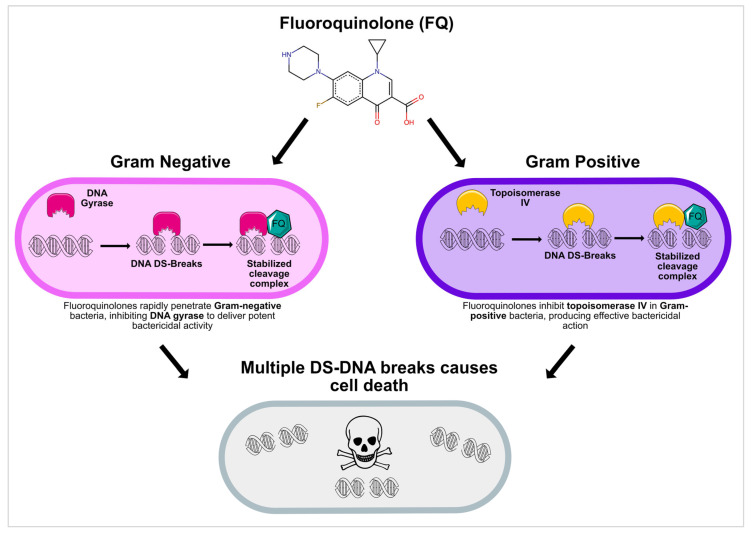
Fluoroquinolones target DNA gyrase in Gram-negative and topoisomerase IV in Gram-positive bacteria, leading to irreversible DNA breaks and cell death. Credit to bioicons.com for the icons in this figure.

FQs exert their antibacterial effects by targeting DNA gyrase and topoisomerase IV, both critical for bacterial DNA replication and transcription. These agents irreversibly stabilize the DNA-enzyme complex, disrupting the progression of the DNA replication fork and leading to the accumulation of double-strand breaks in the bacterial chromosome (Figure 1) [14]. The formation of ternary complexes, drug-enzyme-DNA, prevents the re-ligation of cleaved DNA strands, resulting in lethal double-strand breaks and bacterial cell death [15,16,17]. In Gram-negative bacteria, DNA gyrase is typically the primary target, whereas topoisomerase IV is more frequently targeted in Gram-positive bacteria [14]. Moreover, FQs also induce the production of reactive oxygen species (ROS), leading to oxidative damage to bacterial DNA, proteins, and lipids, exacerbating the effects of DNA strand breaks [18,19]. These mechanisms merely suppress bacterial growth without causing immediate cell death, unlike FQs, which induce lethal DNA damage and oxidative stress, resulting in rapid bacterial killing [20].

### 2.2. Fluoroquinolone Resistance

Resistance to FQs is multifactorial and continues to rise globally, limiting their clinical utility. The most prevalent mechanism involves mutations in the quinolone resistance-determining regions (QRDRs) of genes encoding DNA gyrase (gyrA, gyrB) and topoisomerase IV (parC, parE) [21]. In Gram-negative bacteria such as E. coli, primary resistance arises from gyrA mutations, often at Ser83, while secondary parC mutations amplify resistance. These strains may also upregulate the AcrAB-TolC efflux pump or lose outer membrane porins, further reducing susceptibility. In Gram-positive organisms such as *S. aureus* and *S. pneumoniae*, resistance stems from mutations in grlA, grlB, and gyrA, with dual mutations driving high-level resistance. Efflux pumps such as NorA and PatAB also contribute. Plasmid-mediated quinolone resistance (PMQR) genes, including qnr, aac(6′)-Ib-cr, qepA, and oqxAB, are common in Gram-negative species and can spread horizontally. These plasmids often carry additional resistance genes, meaning that even the use of unrelated antibiotics can help maintain FQ resistance. Notably, some resistance mutations impose little to no fitness cost, allowing resistant strains to persist even without FQ exposure [21].

### 2.3. Pharmacokinetics

With generally high oral absorption and bioavailability, oral administration of FQ is extremely effective [22]. Newer-generation FQs exhibit excellent oral bioavailability, typically exceeding 85%, whereas ciprofloxacin, a second-generation FQ, has a reported oral bioavailability ranging from 55% to 88% [23,24]. They exhibit extensive tissue penetration, with volumes of distribution greater than 1.5 L/kg and tissue concentration often exceeding that of plasma [22]. Peak plasma concentrations are typically attained within two hours of oral administration, with elimination half-lives ranging from 6 to 12 h, allowing for once-daily dosing in most patients with normal renal function [22,23]. FQs are metabolized differently based on the agent. For example, while levofloxacin is largely excreted unchanged in the urine, requiring dosage adjustments in patients with renal impairment, moxifloxacin undergoes hepatic metabolism [23]. Systemic administration of FQs allows for broader tissue distribution but at lower concentrations. This is advantageous for treating widespread infections by ensuring extensive tissue penetration. However, it also increases the risk of systemic adverse effects and the potential for antibiotic resistance. Conversely, topical formulations deliver high local concentrations, making them highly effective for treating localized infections such as external otitis and mild skin infections. This approach minimizes systemic side effects and reduces the risk of resistance development [7,25].

### 2.4. Anti-Inflammatory Properties

While the anti-inflammatory properties of FQs are evident in various studies, their clinical application as anti-inflammatory agents remains limited. FQs have been shown to reduce the production of key pro-inflammatory cytokines, including interleukin-1β (IL-1β), tumor necrosis factor-alpha (TNF-α), and interleukin-6 (IL-6). For instance, levofloxacin has been demonstrated to decrease these cytokines in human peripheral blood mononuclear cells [26]. A study found that ciprofloxacin and levofloxacin can inhibit the activation of the Toll-like receptor 4 (TLR4) and nuclear factor kappa-light-chain-enhancer of activated B cells (NF-κB) pathway in microglial cells [27]. This suppression leads to a decrease in the release of pro-inflammatory cytokines, suggesting a mechanism for their anti-inflammatory effects. They have been reported to inhibit the production of interleukin-1 and TNF-α while enhancing the synthesis of colony-stimulating factors, which play a role in hematopoiesis [28]. Overall, FQs exhibit anti-inflammatory effects through the inhibition of pro-inflammatory cytokines, suppression of key inflammatory signaling pathways, and modulation of immune responses. However, further clinical research is necessary to fully understand and harness these properties in therapeutic settings.

## 3. Clinical Indications in Dermatology

FQs are infrequently prescribed by dermatologists compared to other antibiotic classes, typically reserved for bacterial SSTIs involving Gram-negative and resistant organisms. Their excellent tissue penetration, broad-spectrum activity, and oral bioavailability make them a practical choice in cases where first-line agents fail. However, their use must be balanced against emerging antimicrobial resistance and safety concerns [29]. Table 2, Table 3 and Table 4 lists infections that can be treated with FQs, either as monotherapy or in combination with other antibiotics, adapted from the 2014 Infectious Diseases Society of America (IDSA) clinical practice guidelines for SSTIs along with updated information from the 2024 IDSA recommendations for the treatment of antimicrobial resistant Gram-negative infections [30,31]. These tables are grouped by clinical context: necrotizing, surgical site, and deep soft tissue infections (Table 2), zoonotic and vector-borne infections (Table 3), and atypical and opportunistic pathogens (Table 4). Delafloxacin, a fourth-generation FQ with U.S. Food and Drug Administration (FDA) approval in 2017 for ABSSSIs, is notably not included as the guidelines were published before its approval for use [12].

### 3.1. Common Skin and Soft Tissue Infections

#### 3.1.1. Cellulitis

Cellulitis, a common bacterial SSTI that presents with localized skin erythema, edema, and tenderness to palpation, is caused predominantly by Gram-positive organisms. It most often occurs in middle-aged and older adults and is typically treated with beta-lactam antibiotics such as cephalexin or dicloxacillin [32]. However, in patients with beta-lactam allergies or suspected Gram-negative involvement, doxycycline with an FQ such as ciprofloxacin for five days serves as a viable alternative [30]. Some studies suggest their efficacy in polymicrobial cellulitis, particularly in cases complicated by anaerobic bacteria or *P. aeruginosa* [33].

#### 3.1.2. Impetigo

Impetigo is a highly transmissible superficial bacterial skin infection, predominantly affecting children, with *S. aureus* and *S. pyogenes* as causative organisms [34]. While mild cases are often managed with topical antibiotics, increasing resistance, particularly to mupirocin and fusidic acid [35], has led to the exploration of novel agents. Ozenoxacin 1% cream, a topical non-fluorinated quinolone, has demonstrated potent bactericidal activity and a favorable safety profile in both pediatric and adult populations. It is FDA-approved for the treatment of non-bullous and bullous impetigo in patients 2 months and older [36]. Ozenoxacin has shown in vitro efficacy against MRSA, excellent skin penetration, and a low propensity for resistance development. Given its twice-daily dosing and short 5-day treatment course, ozenoxacin offers a practical and effective option for managing localized impetigo, helping reduce reliance on systemic antibiotics and supporting antimicrobial stewardship [37].

#### 3.1.3. Pseudomonas Infections

FQs, particularly ciprofloxacin, exhibit high activity against *P. aeruginosa* and are frequently used in persistent *Pseudomonas* folliculitis, colloquially known as hot tub folliculitis [29]. Ciprofloxacin is also used for Gram-negative toe web infections and severe cases of *Pseudomonas* nail infections [38,39]. Topical nadifloxacin cream has shown efficacy with off-label use for *Pseudomonas* nail infections [40]. In more severe cases, such as diabetic foot infections, FQs are often incorporated into multidrug regimens to target resistant *Pseudomonas* strains [41]. Their excellent tissue penetration makes them a strong candidate in ischemic diabetic foot ulcers, where antibiotic delivery is limited [30].

#### 3.1.4. Necrotizing Fasciitis

FQs are generally not first-line agents for necrotizing fasciitis—positioned behind broad-spectrum antibiotics such as vancomycin or linezolid plus piperacillin-tazobactam, a carbapenem, or ceftriaxone and metronidazole—but they may be used in combination therapy when Gram-negative and anaerobic involvement is suspected [30]. Given their potent deep tissue penetration, FQs like moxifloxacin have been considered in specific cases, particularly in polymicrobial infections involving *Pseudomonas* and *Klebsiella* species [42]. Ciprofloxacin can also be given 400 mg every 12 h IV in combination with doxycycline for the treatment of necrotizing infections of the skin [30]. Examples of regimens used in necrotizing fasciitis and deep surgical infections, including combination approaches for polymicrobial pathogens, are shown in Table 2.

#### 3.1.5. Diabetic Foot Infections

Patients with diabetes are at increased risk for multidrug-resistant infections, particularly those involving *P. aeruginosa* or other Gram-negative bacilli [29]. Ciprofloxacin and moxifloxacin have demonstrated efficacy in deep or chronic diabetic foot ulcers, especially in patients with ischemic limb disease, where alternative agents may have difficulty penetrating tissue [43].

### 3.2. Atypical and Opportunistic Infections

#### 3.2.1. Atypical Mycobacterial Infections

FQs have shown activity against certain atypical mycobacteria, including *Mycobacterium marinum*, making them an adjunctive option in cutaneous mycobacterial infections [44]. Their use is particularly relevant in immunocompromised hosts, where infections can be severe and refractory to standard therapies [29].

#### 3.2.2. Gram-Negative Bacilli in Immunocompromised Hosts

Immunosuppressed patients, including solid organ transplant recipients, are at higher risk for multidrug-resistant Gram-negative infections affecting the skin. FQs are sometimes used as prophylactic agents in these populations to prevent *Pseudomonas* infections [45]. Selected prophylactic and therapeutic uses of FQs for atypical and opportunistic pathogens, including Gram-negative SSTIs, are shown in Table 3.

### 3.3. Special Dermatological Conditions

#### 3.3.1. Burn Wound Infections

FQs, particularly intravenous ciprofloxacin, have demonstrated efficacy in burn wound infections caused by *P. aeruginosa* and *Acinetobacter* species. Their use is particularly valuable in severely burned or immunocompromised patients, where Gram-negative infections can be life-threatening [46].

#### 3.3.2. Acne Vulgaris

FQs are not first-line antibiotic treatments for acne vulgaris [29]; the prescribing of oral tetracyclines accounts for about three-fourths of all prescriptions [47]. FQs have been utilized in rare cases of Gram-negative folliculitis, particularly in patients with long-term antibiotic exposure leading to resistant cutaneous infections [29].

#### 3.3.3. Surgical Prophylaxis

Routine use of FQs for surgical prophylaxis is not recommended due to resistance concerns and safety risks. However, in select high-risk cases, such as Mohs surgery in immunosuppressed patients, their use has been considered [30].

#### 3.3.4. Hidradenitis Suppurativa (HS)

Moxifloxacin, an FQ, used in combination with rifampin and metronidazole, has shown benefits in softening hypertrophic scars and reduction in pain, erythema, and drainage in 6/6, 8/10, and 2/12 patients with Hurley stage I, II, and III HS, respectively [48]. For patients with more advanced HS (Hurley stages II and III), a combination of ofloxacin and clindamycin is recommended [49].

#### 3.3.5. Zoonotic and Vector-Borne Infections

Zoonotic and vector-borne dermatologic infections treatable with FQs, such as anthrax, tularemia, and glanders, are detailed in Table 4.

## 4. Safety Profile and Risk Management

### 4.1. Adverse Effects (Table 5)

#### 4.1.1. Tendinopathy and Tendon Rupture

One of the most well-documented risks of FQs is tendinopathy, particularly affecting the Achilles tendon [50]. These odds are significantly elevated in patients over 60 years of age (OR 8.3 vs. 1.6) and those taking concomitant oral glucocorticoids (OR 9.1 vs. 3.2) [51]. The mechanism involves collagen degradation via matrix metalloproteinases, leading to weakened tendons and an increased likelihood of rupture [52].

Factors that increase the risk of FQ-induced tendinopathy include advanced age, corticosteroid therapy, renal failure, diabetes mellitus, and a history of musculoskeletal disorders [53]. Patients should be advised to report any new tendon or joint pain promptly. If symptoms develop, FQ therapy should be immediately discontinued, and the patient should be referred for imaging, orthopedic evaluation, and initiation of physical therapy [54]. Additionally, the patient should be advised to avoid physical activity to prevent further tendon damage [55]. Tendinosis usually resolves within several weeks, often by two months after cessation of FQ therapy [53]. Early recognition of symptoms, along with prompt discontinuation of the medication and appropriate supportive care, may prevent tendon rupture [54].

#### 4.1.2. QT Interval Prolongation

FQs indirectly block human delayed-rectifier potassium channels, which can prolong the QT interval and increase the risk of torsades de pointes, a potentially fatal arrhythmia [30,56]. Risk factors include hypokalemia, bradycardia, increased age, and concurrent use of other QT-prolonging agents (e.g., macrolides and antipsychotics). These patients should obtain a baseline 12-lead ECG to assess QTc interval, with ongoing monitoring through comparison with subsequent ECGs [57]. The normal values for QTc range between 350 to 450 ms for adult men and 360 to 460 ms for adult women [58]. If the QTc interval exceeds 500 ms, the FQ should be discontinued, as reviews of studies and expert opinions highlight the increased risk of torsades de pointes [59]. Although QTc prolongation criteria have been recommended, there is no firmly established threshold considered free of proarrhythmic risk [60].

#### 4.1.3. Photosensitivity

There are two types of photosensitivity reactions associated with FQ use. Phototoxicity is the more common reaction and occurs when FQs absorb UV-A light, leading to the generation of reactive oxygen species (ROS). These ROS cause direct cellular damage, including protein oxidation, lipid peroxidation, and DNA strand breaks [61]. They present as exaggerated sunburns as the skin becomes erythematous and edematous, and may develop bullae, usually confined to sun-exposed areas such as the face, neck, and arms.

Another type of reaction is photoallergy, an immunologically mediated reaction that occurs upon exposure to UV light that causes FQs to form UV-induced protein-drug complexes with skin proteins, creating new antigens. These antigens may trigger a delayed hypersensitivity reaction involving T cells [62]. Photoallergic reactions resemble eczematous dermatitis and, unlike phototoxic reactions, can spread beyond sun-exposed areas. The onset of these reactions differs, as phototoxic reactions typically occur within minutes to hours after UV exposure. Meanwhile, photoallergic reactions have a delayed onset, typically occurring 24–72 h after UV exposure.

FQs have varying degrees of phototoxicity assessed by measuring the minimal erythema dose (MED), the lowest dose of UV radiation that causes erythema of the skin. A study comparing sitafloxacin, sparfloxacin, enoxacin, and levofloxacin demonstrated varying degrees of phototoxic potential. Sparfloxacin exhibited a severe reduction in MED, indicating high phototoxic potential and less UV exposure needed to cause sunburn. Enoxacin showed moderate phototoxicity while sitafloxacin had mild phototoxic effects. Levofloxacin did not demonstrate any phototoxicity [63]. The variability is influenced by their chemical structure. For example, FQs with a halogen atom at position 8, such as lomefloxacin and sparfloxacin, were found to have higher phototoxic potential [64]. Severe phototoxic reactions, associated with ciprofloxacin and lomefloxacin, lead to intense sunburns and blistering after UV-A exposure [29]. A study found that FQs can generate singlet oxygen and superoxide anion upon exposure to UV light [61]. Another study demonstrated UV-A radiation enhancement of the cytotoxic effects of lomefloxacin, which is associated with decreased superoxide dismutase (SOD) activity and increased catalase (CAT) and glutathione peroxidase (GPx) activities, indicating oxidative stress [65]. This suggests ROS-mediated damage is a key factor in phototoxicity with FQs. There is a theoretical risk in the release of fluoride ions during the photodecomposition of FQs as a mechanism of their potent phototoxic properties, but there is no direct evidence linking fluoride ions to hypersensitivity reactions.

To mitigate this complication, patients should be advised to wear protective clothing, avoid sun exposure and artificial UV radiation sources, and use broad-spectrum sunscreens. If patients develop photosensitivity reactions, such as erythema, blistering, or edema, the FQ should be discontinued immediately to avoid further phototoxic damage [66]. These symptoms should be managed with topical corticosteroids to reduce inflammation, and in severe cases, systemic corticosteroids may be considered [67].

#### 4.1.4. Gastrointestinal and Neurological Effects

Common adverse effects include nausea, vomiting, dizziness, and headaches. Rare but severe neuropsychiatric effects include hallucinations, confusion, and seizures, particularly in elderly patients [68]. In recent years, chronic symptoms affecting musculoskeletal, neurological, and cognitive functions have been linked to FQ use. Although rare, fluoroquinolone-associated disability (FQAD) has led to increased regulatory scrutiny and legal actions [69].

#### 4.1.5. Hypo- and Hyperglycemia

FQs interfere with glucose homeostasis, leading to hypoglycemia in insulin-dependent diabetics and hyperglycemia in non-diabetics [70]. Research has indicated that fluoroquinolones may lower blood glucose levels by enhancing insulin secretion. This appears to occur through the inhibition of ATP-sensitive potassium channels in pancreatic beta cells [71,72]. However, the clinical relevance of this hypoglycemic effect likely varies depending on the individual’s ability to maintain glucose homeostasis. In contrast, the exact cause of fluoroquinolone-associated hyperglycemia remains unclear, though one possible explanation is excessive drug accumulation in patients with impaired kidney function due to inadequate dose adjustment [73].

#### 4.1.6. Aortic Aneurysm and Aortic Dissection

FQ usage was found to be associated with aortic aneurysms and aortic dissection [74]. This risk is particularly important in dermatology patients with underlying connective tissue disorders (e.g., pseudoxanthoma elasticum, Ehlers-Danlos syndrome, Marfan syndrome) who might be at higher risk. A patient history of obstructions or aneurysms of the aorta or other blood vessels, hypertension, genetic disorders that involve blood vessel changes, and advanced age may warrant alternative antibiotics. Patients should be counseled to watch for sudden onset of chest, abdominal, or back pain [75].

### 4.2. Box Warnings and Regulatory Considerations

Given the safety concerns and regulations surrounding FQs, their use in dermatology should be selective. Prescribers must weigh clinical necessity against the risk of long-term toxicity. Below are key strategies to mitigate adverse effects while preserving efficacy.

The FDA has strengthened its warnings on FQs, particularly regarding tendon rupture, neurotoxicity, and glucose dysregulation [76]. Tendinopathy related to FQ use has been observed to develop anytime from several hours to several months after starting the medication, with the median onset occurring around six days [77]. Peripheral neuropathy has a relative incidence that is highest while the patient is taking an oral FQ (adjusted incidence rate ratio [aIRR], 1.47; 95% CI, 1.13–1.92) and remains significantly increased up to 180 days after FQ exposure (aIRR, 1.25; 95% CI, 1.03–1.51) [78]. Hypoglycemia can occur within hours to days of FQ administration, particularly in patients with diabetes [79,80]. Dermatologists must carefully weigh these risks when considering FQ therapy.

### 4.3. Risk Mitigation Strategies

Given the serious safety concerns associated with FQs, their use in dermatology should be selective. The key to safe prescribing lies in careful patient selection, dose optimization, patient education, and close monitoring [81].

First, patient screening is critical. FQs should be avoided in those with a history of tendon rupture or chronic musculoskeletal disorders, and those on concurrent corticosteroids, which significantly heighten tendon toxicity risk [82]. Patients with cardiac conditions should also be assessed, as these antibiotics can prolong the QT interval and increase the risk of fatal arrhythmias, particularly in those taking other QT-prolonging medications (e.g., ondansetron, haloperidol, citalopram, erythromycin) [81,83]. In diabetic patients, FQs have been linked to glucose dysregulation, requiring closer monitoring [84].

Dosing and route selection also matter. When possible, topical formulations should be used over oral to minimize systemic exposure, as seen with ciprofloxacin ear drops for external otitis [46]. When oral therapy is necessary, the shortest effective course should be used, as prolonged exposure dramatically increases the risk of tendinopathy, neurotoxicity, and dysglycemia [85].

Educating patients is essential. They should be explicitly warned about the risk of tendon rupture, nerve damage, and phototoxicity. If new-onset joint pain, numbness, or burning sensations occur, the medication should be discontinued immediately. Patients should also be counseled on strict sun protection, especially when taking ciprofloxacin or lomefloxacin, as these drugs significantly increase UV sensitivity [82].

Active monitoring can prevent long-term disability. High-risk patients should be followed closely, and any signs of musculoskeletal, neurologic, or cardiac toxicity should prompt immediate discontinuation [85]. FQ toxicity is often irreversible, making early recognition crucial [86]. Clinicians should also remain updated on emerging safety data, as post-marketing surveillance continues to uncover new risks [81].

Ultimately, FQs should be reserved for cases where their benefits outweigh their risks. Dermatologists must be mindful of growing antimicrobial resistance and regulatory restrictions, ensuring that these drugs are used only when necessary.

## 5. Antibiotic Stewardship in Dermatology

Dermatologists account for an estimated 5.4 million antibiotic prescriptions annually, making them among the highest outpatient antibiotic prescribers by specialty [87]. However, fluoroquinolones are relatively rarely used in dermatology, primarily reserved for more severe SSTIs [88]. Stewardship efforts remain essential across all antibiotic classes, including FQs, given their potential for resistance and adverse effects.

Resistance patterns to FQs have been observed both in the U.S. and globally, particularly in *P. aeruginosa*, *E. coli*, and *S. aureus* [89,90]. Bacteria develop fluoroquinolone resistance through multiple mechanisms, including mutations in genes encoding DNA gyrase and topoisomerase IV (the primary FQ targets), upregulation of efflux pumps, decreased membrane permeability, and plasmid-mediated resistance [21]. Optimal use of FQs includes culture-guided therapy, appropriate dosing and duration, and regular reassessment to enable de-escalation or discontinuation when indicated. Pre-prescription approval strategies, such as requiring authorization before prescribing FQs and reviewing prescriptions post-prescription to ensure appropriateness, have been shown to reduce FQ usage [91]. Additionally, implementing syndrome-specific interventions can reduce unnecessary FQ use [92]. The FDA has issued multiple safety warnings regarding FQs and recommends avoiding their use for uncomplicated infections when effective alternative treatments are available [93]. The use of FQs should be guided by susceptibility date and clinical necessity. Dermatologists play a key role in antibiotic stewardship by educating patients, promoting evidence-based prescribing, using narrow-spectrum agents when feasible, and contributing to institutional stewardship programs [30,94].

## 6. Special Patient Populations and Considerations

The use of FQs in dermatology necessitates careful consideration in specific patient populations due to potential adverse effects and varying pharmacokinetics.

### 6.1. Geriatric Patients

#### 6.1.1. Dosing Adjustments

In elderly patients, renal function often declines with age, leading to reduced clearance of renally excreted drugs like FQs. This necessitates dose adjustments to prevent accumulation and toxicity. Additionally, polypharmacy is common in this population, increasing the risk of drug–drug interactions. For instance, FQs can inhibit cytochrome P450 enzymes, potentially leading to elevated levels of co-administered drugs metabolized by these pathways. Careful medication reconciliation and monitoring are essential to mitigate these risks [95].

Elderly patients are at an increased risk for FQ-associated tendinopathy and tendon rupture, particularly those concurrently using corticosteroids. The risk is further heightened in individuals with chronic renal failure. Moreover, FQs have been associated with QT interval prolongation, which can precipitate life-threatening arrhythmias, especially in patients with existing cardiac conditions or those taking other QT-prolonging medications. Therefore, these antibiotics should be used with caution in this population [96,97].

#### 6.1.2. Medication Adherence

Elderly patients, particularly those reliant on caregivers, may face challenges with medication adherence, especially with complex dosing regimens like four times daily (i.e., QID). Simplifying dosing schedules, providing clear instructions, and involving caregivers in the treatment plan can enhance adherence and therapeutic outcomes.

### 6.2. Patients with Rheumatologic–Dermatologic Diseases

Patients with rheumatologic conditions such as psoriatic arthritis and rheumatoid arthritis inherently have an increased risk of tendon inflammation. The use of FQs in these individuals requires additional caution due to the potential exacerbation of tendinopathy, necessitating vigilant monitoring for tendon-related symptoms. Additionally, the concurrent use of corticosteroids and FQs has been associated with an increased risk of tendon rupture. Clinicians should weigh the benefits against the risks and consider alternative antibiotics when appropriate.

### 6.3. Immunocompromised Patients

Immunocompromised individuals are more susceptible to atypical and severe infections. While FQs offer broad-spectrum coverage, their use should be guided by culture and sensitivity results to ensure efficacy and reduce the risk of resistance.

In immunocompromised patients, the need for broad-spectrum antibiotic coverage must be balanced against the potential for adverse effects. Close monitoring for toxicity and therapeutic efficacy is crucial in this population.

### 6.4. Pediatric Patients

Traditionally, FQs have been contraindicated in children due to concerns about articular cartilage damage. However, some experts, including the American Academy of Pediatrics, suggest that these antibiotics can be considered as second-line agents in specific situations where no reasonable alternatives exist [98]. Notably, a national retrospective cohort study found no increased risk of Achilles tendinopathy in children under eight years of age treated with FQs for pneumonia, indicating that the risk may be lower than previously thought [99].

### 6.5. Pregnant Populations

The use of FQs during pregnancy has been a subject of debate, despite the lack of teratogenic effects observed in animal studies [100,101]. Human studies have not consistently demonstrated an increased risk of major malformations [102,103]. Regardless, FQs are typically avoided during pregnancy unless no safer alternatives are available.

### 6.6. Patients with Seizure Disorders

FQs are thought to inhibit GABA-A receptors in the CNS, increasing excitatory signaling and lowering the seizure threshold [104]. This is particularly true in patients with predisposing factors such as epilepsy, severe cerebral atherosclerosis, or other CNS disorders [105]. The FDA considers FQs to be a relative contraindication in patients with known or suspected CNS disorders [106,107].

## 7. Discussion

### 7.1. Clinical Decision-Making

FQs remain an important treatment option in dermatology for SSTIs, particularly those involving Gram-negative or resistant organisms [3,30]. Their broad spectrum of activity, high oral bioavailability, and tissue penetration make them especially beneficial in complex infections, including diabetic foot wounds and Pseudomonas-related conditions. However, when prescribing FQs, dermatologists must navigate a delicate balance between clinical efficacy and the potential for significant adverse effects, such as tendinopathy, QT interval prolongation, and neurotoxicity, as well as concerns over growing antimicrobial resistance [4].

From a practical standpoint, clinicians should primarily consider FQs for patients with documented Gram-negative infections or when standard first-line agents fail or are contraindicated. In selecting a specific FQ, clinicians must account for local resistance patterns, individual patient comorbidities, and potential drug–drug interactions, especially in older adults with multiple medications. In many cases, topical FQs provide a targeted, high local concentration with reduced systemic exposure, thus mitigating some of the most worrisome adverse effects [7,25]. Ultimately, clinical decision-making must incorporate not only antimicrobial coverage but also patient-related factors that influence safety, tolerability, and compliance.

### 7.2. Clinical Pearls

1. Screen for Risk Factors: Identify patients at higher risk of FQ-related complications, such as the elderly, those with tendon disorders, aortic aneurysm risk, or those on corticosteroids, and consider alternative therapies if possible;
2. Shorten Treatment Duration: Whenever clinically feasible, opt for the minimum effective course to limit adverse events and reduce the likelihood of resistance;
3. Monitor for Toxicity: Advise patients to watch for early signs of tendon pain, neuropathy, or cardiac symptoms and to stop therapy immediately if they develop these symptoms;
4. Leverage Topical Formulations: In localized infections amenable to topical therapy (e.g., chronic otitis externa or mild wound infections), a topical FQ can achieve high local concentrations while minimizing systemic effects [7];
5. Engage in Stewardship: Confirm bacterial pathogens with cultures and tailor therapy to sensitivity results, collaborating with infectious disease specialists or stewardship teams as needed.

### 7.3. Controversies and Debates

Debate continues over the role of FQs as first-line vs. second-line agents in dermatology. Regulatory bodies, including the FDA, have issued multiple safety communications highlighting FQ-associated tendon rupture, neuropathy, and other severe side effects [76]. Nevertheless, real-world prescribing patterns suggest that FQs remain an attractive choice in scenarios demanding broad-spectrum coverage, especially in severe or polymicrobial SSTIs where swift, high-efficacy treatment is paramount [29,85]. The tension lies between recognized toxicities and the clinical benefits in acute, high-stakes infections, such as necrotizing fasciitis or complicated diabetic foot ulcers.

Another point of debate is whether FQs accelerate resistance more rapidly than other antibiotic classes due to their frequent use in both inpatient and outpatient settings. Dermatologists, who rank among the highest prescribers of outpatient antibiotics, are under increasing pressure to adhere to antibiotic stewardship principles to help curb resistance. Striking the right balance between the need for immediate, effective therapy and the long-term implications of emerging resistance remains a core controversy surrounding FQ use [87,88,89,90,108].

### 7.4. Limitations of Current Evidence

Despite the extensive literature on FQ pharmacology and its role in infectious diseases, large-scale trials focusing specifically on dermatologic populations are limited [3,108]. Much of the evidence supporting FQ use in skin infections has been extrapolated from broader clinical studies in internal medicine or smaller case series in dermatology. Consequently, knowledge gaps persist regarding optimal dosing strategies, comparative effectiveness vs. other antibiotic classes, and the long-term safety of FQs for chronic dermatologic conditions. Additionally, antimicrobial resistance patterns for dermatologic practice outside the US are limited. Future research is necessary to better understand global resistance patterns in dermatologic practice.

Moreover, few robust guidelines address the precise positioning of FQs in managing specific skin conditions beyond established indications, such as Pseudomonas infections and complicated SSTIs [30]. Additional prospective studies—especially those comparing FQs with other antibiotic classes in dermatology-specific cohorts—would provide clearer guidance on patient selection, duration of therapy, and risk mitigation strategies.

Limitations of this literature review include the lack of a standardized quality assessment for peer-reviewed articles that were cited. Additionally, this review relies largely on studies not specific to dermatology given the available literature. For information from government regulatory agencies, only sources from the United States government were included given the primary authors’ familiarity.

## 8. Key Takeaways for Dermatologists

FQs have unique advantages in dermatology, including broad-spectrum coverage, high oral bioavailability, and potent activity against problematic Gram-negative organisms. These attributes can be crucial for managing severe or treatment-resistant SSTIs, diabetic foot infections, and certain atypical infections. However, the risk of serious adverse effects, including tendinopathy, QT interval prolongation, neurotoxicity, and photosensitivity, requires that dermatologists exercise heightened vigilance when selecting and prescribing FQs.

Antibiotic stewardship principles should guide every step of FQ use, from deciding whether an antibiotic is truly necessary to choosing the narrowest effective agent and the shortest feasible duration of therapy [87,88,108]. In addition, dermatologists must remain updated on evolving resistance patterns and regulatory advisories that may alter the risk-benefit profile of FQs over time.

## 9. Future Directions

### 9.1. Research Gaps and Innovation Priorities

The evolving landscape of dermatologic therapeutics necessitates continuous research to optimize FQ use while mitigating risks. Current literature on FQs in dermatology is limited, highlighting the need for prospective studies to evaluate their efficacy and safety in treating SSTIs. Such studies would inform evidence-based guidelines and stewardship interventions, promoting judicious use of FQs in dermatologic practice [108].

The development of novel FQ derivatives aims to enhance antimicrobial efficacy while reducing adverse effects. Research into these derivatives could yield agents with improved safety profiles, expanding therapeutic options in dermatology.

Advancements in drug delivery systems, such as extended-release and targeted-delivery formulations, hold promise for improving the therapeutic index of FQs. These innovations could enhance local drug concentrations, minimize systemic exposure, and reduce the risk of resistance development [109].

### 9.2. Addressing Resistance and Personalization in Antibiotic Therapy

Ongoing surveillance of resistance patterns is crucial for informing clinical practice. Regular updates to treatment guidelines based on surveillance data ensure that therapeutic strategies remain effective against evolving pathogens [110,111].

Integrating pharmacogenomic data and biomarkers into clinical decision-making can facilitate personalized antibiotic therapy. This approach allows for tailoring antibiotic selection to individual patient profiles, potentially enhancing efficacy and reducing adverse reactions [112].

## 10. Conclusions

FQs remain valuable therapeutic tools in dermatology, particularly for challenging infections requiring broad-spectrum coverage or deep tissue penetration. Yet, their severe adverse events and the mounting evidence of fluoroquinolone resistance underscore the importance of judicious use. Addressing research gaps, advancing drug formulations, monitoring resistance trends, and integrating personalized medicine will be pivotal to optimizing their role in dermatological practice. A measured, evidence-based approach—grounded in patient-specific considerations, stewardship principles, and continuous monitoring—enables dermatologists to harness the benefits of this antibiotic class while minimizing risks. Through prudent prescribing and ongoing education, fluoroquinolones can continue to play a pivotal, albeit carefully regulated, role in modern dermatologic practice.

## Figures and Tables

**Table 1:** Classification of fluoroquinolones by generation.

Generation	Agents	Half Life	Comments
1st	Nalidixic acid	4–6 h	Prototypical quinolone; limited use in dermatology
2nd	Ciprofloxacin, Norfloxacin, Ofloxacin, Nadifloxacin (topical)	6–8 h	Enhanced Gram-negative coverage; common in SSTIs; Used off-label topically for skin infections like *Pseudomonas* nail (nadifloxacin)
3rd	Levofloxacin, Moxifloxacin	8–10 h	Expanded Gram-positive coverage; once-daily dosing
4th	Delafloxacin, Trovafloxacin	10–12 h	Broad-spectrum, including MRSA and anaerobes; acidic pH activity (delafloxacin)

**Table 2:** Fluoroquinolone agents and dosing in necrotizing, surgical site, and deep soft tissue infections.

Indication	Regimen(s)	Target(s)	Evidence	Notes
Incisional surgical site infections following operations on the axilla, perineum, or female genital tract	*Combination therapy* Ciprofloxacin 400 mg IV every 12 h or 750 mg PO every 12 h + metronidazole 500 mg every 8 h IV Levofloxacin 750 mg IV every 24 h + metronidazole 500 mg every 8 h IV *Surgery of the axilla or the perineum* ^ Metronidazole 500 mg every 8 h IV + Ciprofloxacin 400 mg IV every 12 h or 750 mg PO every 12 h Levofloxacin 750 mg every 24 h IV po	Gram-negative bacteria and anaerobes	Strong, low	
Treatment of necrotizing infections of the skin	*Antimicrobial Agent for Patients with Severe Penicillin Hypersensitivity* Clindamycin or metronidazole * with an aminoglycoside or fluoroquinolone	Mixed Infections		
Treatment of necrotizing infections of the skin	Doxycycline (100 mg every 12 h IV) plus Ciprofloxacin (400 mg every 12 h IV) or Ceftriaxone (1 to 2 g every 24 h IV)	*Aeromonas hydrophila*		Not recommended for children, but may need to use in life-threatening situations.

^ May also need to cover for methicillin-resistant *S. aureus* with vancomycin 15 mg/kg every 12 h. * If *Staphylococcus* is present or suspected, add an appropriate agent.

**Table 3:** Fluoroquinolone agents, dosing in in atypical and opportunistic pathogens.

Indication	Regimen(s)	Target(s)	Evidence	Notes
Patients with SSTIs during the initial episode of fever and neutropenia	Ciprofloxacin and amoxicillin-clavulanate PO for low-risk patients Levofloxacin therapy 750 mg PO daily may be considered For 7–14 days			If fluoroquinolones are used for prophylaxis, broad-spectrum β-lactam antibiotics should be used for empiric therapy
Cutaneous *Nocardia*	Extended-spectrum fluoroquinolones (e.g., moxifloxacin) for 6–24 months	*Nocardia farcinica, Nocardia brasiliensis,* and other *Nocardia* species		Combine with other agents for patients with severe infections or profound/lasting immunodeficiency

**Table 4:** Fluoroquinolone agents and dosing in zoonotic and vector-borne infections.

Indication	Regimen(s)	Target(s)	Evidence	Notes
Infected animal bite-related wounds	Ciprofloxacin 500–750 mg PO bid or 400 mg IV every 12 h Levofloxacin 750 mg PO or IV daily Moxifloxacin 400 mg po or IV daily	Good activity against *Pasturella multocida*; lacks activity against MRSA and some anaerobes	Strong, moderate	Moxifloxacin may be used as monotherapy; effective for anaerobes as well Can be combined with metronidazole for human bites (moxifloxacin may be used as monotherapy) if there is a history of hypersensitivity to β-lactam antibiotics
Cutaneous anthrax	Ciprofloxacin 500 mg PO bid or levofloxacin 500 mg IV/PO every 24 h × 60 days is recommended for bioterrorism cases because of presumed aerosol exposure	*Bacillus anthracis*	Strong, low	PO vs. IV therapy and dosage are dependent on the severity of the illness (estimated by the amount of edema) Gatifloxacin or moxifloxacin is also likely to be effective
Erysipeloid	Fluoroquinolones	*Erysipelothrix rhusiopathiae*		Option for those with penicillin intolerance
Glanders	Ciprofloxacin 400 mg IV every 8 h or 750 mg PO every 12 h	*Burkholderia mallei*	Strong, low	Type of fluoroquinolone based on 2014 IDSA guidelines for skin and soft tissue infections Dosage based on 2024 IDSA guidelines for the treatment of antimicrobial-resistant gram-negative infections
Bubonic plague	Ciprofloxacin for 10–14 days Other fluoroquinolones may be effective	*Yersinia pestis*		Evidence is based on in vitro susceptibilities and murine models
Tularemia	Levofloxacin 500 mg PO daily or ciprofloxacin 750 mg PO bid for at least 14 days	*Francisella tularensis*		For cases of mild to moderate illness

**Table 5:** Adverse effects and safety warnings associated with fluoroquinolones and management recommendations.

Adverse Effect	Clinical Features/Risk Factors	Management Recommendations
Tendinopathy and Tendon Rupture	Achilles tendon is most affected; risk factors: age > 60, corticosteroids, renal failure, diabetes, musculoskeletal disorders	Immediately discontinue FQ; orthopedic referral, imaging, physical therapy; avoid physical activity
QT Interval Prolongation	Risk factors: hypokalemia, bradycardia, increased age, concurrent QT-prolonging drugs (macrolides, antipsychotics)	Baseline and follow-up ECG; discontinue FQ if QTc > 500 ms
Photosensitivity	Severe sunburn, erythema, blistering, edema (notably ciprofloxacin, lomefloxacin)	Avoid sun exposure; use protective clothing and broad-spectrum sunscreen; topical/systemic corticosteroids; discontinue FQ
Gastrointestinal and Neurological	Nausea, vomiting, dizziness, headaches; rare severe effects: hallucinations, confusion, seizures (especially elderly); chronic FQAD symptoms	Symptomatic management; discontinue FQ if severe neuropsychiatric symptoms occur
Hypo- and Hyperglycemia	Glucose homeostasis disruption; hypoglycemia (insulin-dependent diabetics), hyperglycemia (non-diabetics)	Monitor blood glucose closely; adjust diabetic medications accordingly
Aortic Aneurysm and Aortic Dissection	Chest, abdominal, or back pain; risk factors: connective tissue disorders (e.g., pseudoxanthoma elasticum, Ehlers-Danlos syndrome, Marfan syndrome), history of obstructions or aneurysms of the aorta or other blood vessels, hypertension, genetic disorders that involve blood vessel changes, and advanced age	Monitor for chest, abdominal, or back pain occurring within two months of starting an FQ

## Data Availability

Not applicable.

## Abbreviations

The following abbreviations are used in this manuscript:

ABSSSI	Acute Bacterial Skin and Skin Structure Infections
DNA	Deoxyribonucleic Acid
ECG	Electrocardiogram
FDA	Food and Drug Administration
FQ	Fluoroquinolone
FQAD	Fluoroquinolone-Associated Disability
IL-1β	Interleukin-1 Beta
IL-6	Interleukin-6
IV	Intravenous
MSSA	Methicillin-Susceptible *Staphylococcus aureus*
MRSA	Methicillin-Resistant *Staphylococcus aureus*
NF-κB	Nuclear Factor Kappa-Light-Chain-Enhancer of Activated B Cells
PO	By Mouth (Per Os)
QT	QT Interval (on Electrocardiogram)
QTc	Corrected QT Interval
SSTI	Skin and Soft Tissue Infection
SSSIs	Skin and Skin Structure Infections
TNF-α	Tumor Necrosis Factor Alpha
TLR4	Toll-Like Receptor 4
